# Reconciling devolution with health financing and public financial management: challenges and policy options for the health sector

**DOI:** 10.1136/bmjgh-2024-015216

**Published:** 2024-05-29

**Authors:** Nirmala Ravishankar, Inke Mathauer, Hélène Barroy, Ileana Vîlcu, Michael Chaitkin, Marie Jeanne Offosse, Pura Angela Co, Angellah Nakyanzi, Boniface Mbuthia, Salomão Lourenço, Halimah Mardani, Joseph Kutzin

**Affiliations:** 1 ThinkWell, Chennai, Tamil Nadu, India; 2 WHO, Geneva, Switzerland; 3 ThinkWell, Geneva, Switzerland; 4 Bill & Melinda Gates Foundation, Seattle, Washington, USA; 5 ThinkWell, Ougadougou, Burkina Faso; 6 ThinkWell, Manila, Philippines; 7 ThinkWell, Kampala, Uganda; 8 Strategic Purchasing Africa Resource Center, Nairobi, Kenya; 9 ThinkWell, Maputo, Mozambique; 10 ThinkWell, Jakarta, Indonesia; 11 Independent Health Financing Specialist, Genolier, Switzerland

**Keywords:** Health economics, Health policy, Health systems

## Abstract

The interplay between devolution, health financing and public financial management processes in health—or the lack of coherence between them—can have profound implications for a country’s progress towards universal health coverage. This paper explores this relationship in seven Asian and African countries (Burkina Faso, Kenya, Mozambique, Nigeria, Uganda, Indonesia and the Philippines), highlighting challenges and suggesting policy solutions. First, subnational governments rely heavily on transfers from central governments, and most are not required to allocate a minimum share of their budget to health. Central governments channelling more funds to subnational governments through conditional grants is a promising way to increase public financing for health. Second, devolution makes it difficult to pool funding across populations by fragmenting them geographically. Greater fiscal equalisation through improved revenue sharing arrangements and, where applicable, using budgetary funds to subsidise the poor in government-financed health insurance schemes could bridge the gap. Third, weak budget planning across levels could be improved by aligning budget structures, building subnational budgeting capacity and strengthening coordination across levels. Fourth, delays in central transfers and complicated procedures for approvals and disbursements stymie expenditure management at subnational levels. Simplifying processes and enhancing visibility over funding flows, including through digitalised information systems, promise to improve expenditure management and oversight in health. Fifth, subnational governments purchase services primarily through line-item budgets. Shifting to practices that link financial allocations with population health needs and facility performance, combined with reforms to grant commensurate autonomy to facilities, has the potential to enable more strategic purchasing.

**Summary box**

- In most countries, reforms to strengthen health financing arrangements and streamline public financial management (PFM) processes to accelerate progress towards universal health coverage (UHC) are happening alongside or against the backdrop of devolution.

- While a lot has been written about devolution in the health sector in low-income and middle-income countries, how devolution interacts with health financing and PFM processes in the health sector has not been explored rigorously.
- Drawing on case studies from seven Asian and African countries, we identify five common topics: (1) heavy reliance of subnational governments on transfers from the national level to deliver health services entrusted to them, (2) territorial fragmentation of health funds, (3) disparate budgeting across levels of government, (4) weak expenditure management at the subnational level and (5) limited strategic purchasing by subnational governments; under each, we discuss the challenges posed by the current state of play.

- For each topic, we also discuss policy options that decision-makers can consider as they design UHC-oriented reforms such as the use of conditional grants to increase local health budgets, strategies for shifting the pooling to higher levels of government to reduce fragmentation, the potential of public subsidies for health coverage schemes to redistribute resources across territories, the scaling up of consistent budgeting approaches between and across levels to provide better alignment between resources and needs and flexibility, PFM adjustments to streamline expenditure management to maximise outputs and approaches to enhance strategic purchasing by subnational governments.

## Introduction

Low-income and middle-income countries (LMICs) around the world are undertaking health financing reforms to raise more funds for health, create large and diverse pools, and ensure that the funds are spent efficiently to progress towards the goal of universal health coverage (UHC).^1^ There is a growing consensus that public financing is necessary for country progress towards UHC,^2 3^ calling for better understanding of public financial management (PFM) processes that govern how public funds are raised, pooled, spent and monitored.^4 5^

In most LMICs, efforts to strengthen health financing arrangements and streamline PFM in the health sector are happening alongside or against the backdrop of decentralisation, which refers to the transfer of authority and responsibility for public functions from the central government to other public entities. Decentralisation can take different forms including de-concentration (involving local offices of central government ministries), delegation (involving semiautonomous public entities) and devolution (involving subnational governments).^6^ In this paper, we focus on devolution, namely the transfer of decision-making powers and public funds from the centre to subnational governments.

Many LMICs have embraced devolution reforms since the 1980s for different reasons,^7–9^ and the literature on devolution in the health sector is vast. This includes analyses of the nature of powers granted to local authorities^10 11^ and studies examining how devolution has affected different health system building blocks.^12^ Several articles have explored the effects of decentralisation on key aspects of health financing including public and private health spending, equity and efficiency.^9 13–15^ Some have also emphasised the links between decentralisation and PFM processes in the health sector.^16–19^ However, the three-way interplay between devolution, health financing and PFM in health has not been explored in detail.

Our paper seeks to fill this gap based on an analysis of information from seven LMICs in Africa and Asia: Burkina Faso, Indonesia, Kenya, Mozambique, Nigeria, the Philippines and Uganda. The countries were selected purposively to reflect geographical diversity and different degrees of devolution (table 1). Case studies for each were undertaken in 2021 and two related synthesis reports were published in 2022.^20–28^ The first report explored how devolution has shaped health financing functions, namely revenue raising (the way funds are raised to finance the provision of health services), pooling (arrangements for accumulating prepaid funds to share the financial risk of seeking care) and purchasing (the allocation of pooled funds to healthcare providers).^29^ The second report focused on how decentralisation—especially devolution—has influenced PFM processes in the health sector, focusing on budget development, expenditure management and budget reporting. Findings from the reports were discussed at a virtual knowledge exchange involving policymakers and analysts from 27 countries in mid-2022.^30^ This paper draws on this body of work to highlight key issues at the nexus of devolution, health financing and PFM, as well as discuss challenges and potential remedies.

**Table 1:** Role of subnational governments in health service delivery

Country	Subnational levels	Service delivery
Burkina Faso (unitary)	Regions and communes (devolution); provinces and districts (de-concentration)	Ministry of Health (MOH) is responsible for policy and planning, and steers policy implementation through regional and district health offices. The district health offices (which are the administrative branch of MOH) oversee district hospital and primary healthcare (PHC) facilities. PHC centres report to the communes where they are located. Regional health offices oversee regional hospitals.
Indonesia (federal)	Provinces and districts/cities	Central government is responsible for overall health policy and overseeing centrally managed tertiary facilities. Provincial governments have authority to develop provincial policies and regulations. They manage provincial hospitals while districts operate district hospitals and PHC facilities.
Kenya (unitary)	Counties, sub-counties and wards	MOH sets policy and manages tertiary care facilities. Counties directly manage service delivery in all primary and secondary care facilities in the public sector and regulate private providers in their jurisdiction.
Mozambique (unitary)	Provinces, districts and municipalities	MOH sets health sector policies and plans. Provinces are responsible for provincial sector plans and operating provincial facilities. Districts manage health service delivery in their jurisdiction.
Nigeria (federal)	States and local governments	The federal MOH oversees tertiary facilities, and funds select health programmes. States manage their own hospitals and oversee PHC services through PHC Boards. Local governments supervise PHC services.
Philippines (unitary)	Regions, provinces, cities and municipalities	The Department of Health sets policies, regulates services and has direct oversight for some tertiary-level hospitals. Local government units (which include all levels listed in the previous column) are responsible for managing and implementing local health programmes, facilities and services.
Uganda (unitary)	Districts	MOH is responsible for policy development and supervision of the sector. Districts are responsible for overseeing delivery of health services.

## Subnational dependence on national transfers

The share of government health expenditure controlled by subnational governments varies across the seven study countries (table 2). Even so, subnational governments play a significant role in allocating resources for the delivery of health services, especially primary and secondary care (table 3). While subnational governments can raise their own revenue, they rely heavily on transfers from central governments to finance their budgets in all the study countries (table 2). This fiscal gap between revenue raising and spending responsibilities is in keeping with global patterns,^16^ and not a problem per se if intergovernmental transfers are adequate and timely (which is discussed in the section below on expenditure management).

**Table 2:** Subnational governments’ role in health spending

Country	Share of subnational and/or local budget that is financed through transfers from the central government	Share of total government health spending that occurs at the subnational and/or local level
Burkina Faso	43% for communes (2018)	75% at the subnational level, which includes both communes and districts (2017)
Indonesia	47% for provinces and 67% for districts (2018)	10% at the provincial level and 36% at the district level (2018)
Kenya	71% for counties (fiscal year (FY) 2019/2020)	50% at county level (FY 2019/2020)
Mozambique	98% for provinces and 99% for districts (2019)	22% at the provincial level and 24% at the district level (2020)
Nigeria	60% for states and 95% for local government areas including federal transfers and state allocations (2019)	29% at the state level, 8% at the local level (not including federal programmes that provide support to primary care facilities) (2016)
Philippines	61% for local government units (LGUs) (2019)	20% at LGU level (2019)
Uganda	100% for districts	39% at district level (between FY 2015/2016 and 2018/2019)

**Table 3:** Role of subnational governments in health financing

Country	Revenue raising	Pooling	Purchasing
Burkina Faso	Central government raises the bulk of the revenue. Communes are responsible for local revenue raising.	Funds are pooled at the central level.	MOH is the main purchaser of services from all levels of public facilities. It pays for staff salaries. Some of its funds are channelled through district health offices to cover additional costs. Communes allocate funds in their budget to cover operating costs and salary costs for ancillary staff and community health workers through input-based budgets at primary health centres. Public facilities levy user fees except for selected maternal, newborn and child health, and family planning services under the Gratuité scheme, for which they receive direct reimbursement from MOH (or via the district health office in the case of PHC centres). Facilities pay for commodities using their revenue from user fees and Gratuité.
Indonesia	Subnational units can raise revenue, but account for a small share. They rely heavily on transfers from the central government.	There is one national pool from the national budget, and several provincial and district pools. Districts and provinces receive earmarked funds for specific health programmes from the central government, which cannot be pooled with their general budget. The national health insurance scheme, Jaminan Kesehatan Nasional (JKN), pools resources at the central level.	Provincial governments purchase services from provincial hospitals, while district governments purchase services from district hospitals and PHC centres. Both fund preventive and promotive health services and receive funds from the central government to implement vertical health programmes. They largely finance these services through input-based budgets, paying for staff salaries, commodities and operations costs. The central government controls tertiary hospitals in the public sector. JKN purchases services from public and private providers. There are user charges at all private and public facilities for non-JKN members (or members who do not identify as such).
Kenya	Central government raises the bulk of the revenue. Counties generate local revenue, but in practice, this remains a small share of the county budget.	Central government pools tax revenue and allocates a share to counties. Counties pool these transfers with local revenue. The National Health Insurance Fund (NHIF) manages additional pools at central level (covering ~26% of the population).	MOH purchases services from tertiary facilities in the public sector. Counties purchase primary and secondary care from public facilities within their jurisdiction, largely through input-based budgets covering staff salaries, commodities and operating costs. This includes community health workers, and prevention and promotion programmes. NHIF purchases services from both public and private providers. NHIF payments to public facilities are a ‘top-up’ over supply-side financing received by the facilities from the country government. NHIF uses a variety of provider payment mechanisms (eg, case-based payments, per diem and capitation) with both public and private health facilities. Secondary and tertiary hospitals in the public sector charge user fees, while services at the primary level are free.
Mozambique	Central government mobilises and allocates the bulk of funds. Revenue generated by districts and provinces represents a small share of the total.	Funds are pooled at the central level by MOH, provincial level and district level.	Central government purchases services from central and specialised hospitals. It pays salaries of health workers at central hospitals and for commodities and capital investments. Provinces purchase services from hospitals located at the provincial level (central specialised, general and provincial), paying for health worker salaries and other costs through input-based budgets. Provinces also pay for community health workers. District health offices purchase services from district and rural hospitals and health centres, paying largely for health worker salaries and operating costs. All public facilities charge user fees.
Nigeria	The federal tier raises the bulk of government revenue. A large share is transferred to subnational levels. One-fifth of state revenue is internally generated, while local governments rely overwhelmingly on federal transfers.	The federal MOH and each state is a pool. Some states also operate health insurance schemes, but these are small, as is the federally administered National Health Insurance Scheme. There is minimal pooling at the local level.	The federal government purchases services from tertiary facilities and pays for commodities for all levels (often with support from donors). It also directly funds portions of the primary care workforce, such as covering the salaries of midwives and community health extension workers. State governments cover costs of state hospitals, as well as salaries of health workers at state and local levels. Some states operate social health insurance schemes where they purchase a range of services from contracted facilities. Some local governments pay for a share of operating costs of primary care facilities. Most public facilities charge user fees.
Philippines	Central government raises bulk of government revenue. LGUs have the authority to raise their own revenue, but most continue to be highly dependent on the fiscal transfers and allocations received from the central government.	DOH and Philippine Health Insurance Corporation (PhilHealth) manage the biggest fund pools for health at the central level. LGUs pool funds received from central government and their own source revenue into the LGU budget.	DOH purchases services from DOH-owned hospitals through line-item budgets and provides some inputs, including commodities and temporary staff for other public facilities. LGUs purchase services from LGU-owned facilities as line-item budgets. PhilHealth purchases services from both public and private facilities. It pays for primary healthcare services through a mix of capitation and case-based payments, while inpatient services are through case-based payments. Hospitals can charge user fees, but public primary care facilities cannot charge user fees.
Uganda	Central government raises most of the revenue. Districts can generate local revenue, but the amount collected is negligible and must be remitted to the central consolidated fund.	Central government pools all domestic resources.	Central government purchases directly from tertiary facilities, and indirectly from primary and secondary public facilities through grants that are allocated to districts, but either cover staff salaries (over which districts have limited control) or flow directly to facilities. It allocates and transfers funds directly to public facilities to cover operating costs. Districts control financing for community health programmes. Districts receive conditional grants from the central government to pay for capital costs associated with infrastructure upgrades for health facilities. They do not pay for any facility costs. There are no user charges in public facilities (except in private wings of public hospitals; this revenue is remitted to the central level).

DOH, Department of Health; LGUs, local government units; MOH, Ministry of Health; PHC, primary healthcare.

Subnational governments receive block grants from the central government that are not earmarked for any sectors in all study countries except Burkina Faso. This gives subnational governments discretion over how they use these funds, which has resulted in low prioritisation of health by some of them as well as wide variation across subnational governments in several study countries. In Kenya, for example, the allocation to health in the county budget ranged from 17% to 37% in 2019. National mandates for subnational governments to allocate a minimum share of their budgets to health are rare; Indonesia is the only study country where they are legally required to set aside at least 10% for health, but compliance remains uneven. In all study countries, central governments are also using conditional grants to increase health spending by subnational governments. Even so, block grants account for the bulk of the funds for subnational governments among the countries that are more devolved, namely Kenya, Nigeria, Indonesia and the Philippines.

A policy implication of the heavy reliance of subnational governments on intergovernmental transfers is that increases in public financing to drive progress towards UHC will likely have to come from central governments. Moreover, how these funds are transferred to subnational governments will dictate how much of it flows to the health sector. Block grants carry the risk that not all subnational governments will invest more in health. To mitigate this risk, a promising policy option is for central governments to increase the use of conditional grants for health and improve how they are designed and enforced to increase total health spending by subnational governments (eg, by using matching requirements).

## Geographical fragmentation of health funding

By design, devolution leads to funds being divided across subnational governments, which creates fragmented pools. This in turn can make it harder to achieve an equitable distribution of resources across territories. Study countries are using different measures to improve fiscal equalisation, which refers to the transfer of funds to and between subnational governments to address regional differences in fiscal capacity and expenditure needs.^31^ Formula-based block grants that allocate resources based on measures of population size, poverty and health needs exist in all study countries except Indonesia, which provides special grants for disadvantaged areas along with Uganda. Despite these grants, which typically are not earmarked for health, per capita government health expenditure across subnational governments remains inequitable in most study countries.

Given that devolution may entrench regional inequalities, there is a need for central governments to improve horizontal revenue sharing rules and fiscal equalisation arrangements. Reconciling multiple considerations informing the design of these complex formulas—such as balancing the needs of health versus other sectors and reallocating resources according to fiscal capacity while also rewarding effort—is not easy.^32 33^ Depending on the context, conditional grants earmarked for health could be a promising way to distribute health funds to specific areas, as is the case in Uganda and Indonesia. Some countries like the Philippines are also considering shifting the pooling function to higher levels of government.

Government-financed health insurance can also serve as a mechanism to improve resource distribution across territories. Four of the countries—Kenya, Indonesia, Nigeria and the Philippines—have such schemes. Indonesia and the Philippines use general budget funds to cover the poor and have achieved high rates of population coverage,^34^ and Kenya and Nigeria are attempting to do the same. Such subsidies have the potential to redistribute government revenues from richer to poorer areas, given that the people in the former are likely to pay more taxes while the latter have more poor people. The larger the share of the population covered through the health insurance scheme, the larger the potential redistributive effect. Whether this redistribution is achieved in practice, however, depends on other factors including the relative demand for healthcare and the availability of services.

## Disparate budgeting across government levels

In all seven countries, subnational levels undertake budgeting in parallel to the central government. Insufficient coordination and information-sharing between levels as well as capacity constraints impede subnational governments from producing credible health budgets. For example, the central government in the Philippines provides capital financing for new facilities, but local governments either cannot or do not consistently budget for the staff (due to caps on hiring personnel and competing priorities), and uncertainty regarding in-kind transfers from the centre for priority programmes hinders effective budgeting at the local level. In Mozambique, the central government adjusts budgeting guidelines annually without offering sufficient training for subnational actors to implement them.

In all seven countries, multiple funding sources with varying conditionality make it difficult for subnational governments to budget comprehensively. This challenge has been documented elsewhere and is often exacerbated by vertical disease programmes.^16 35^ Funding amounts are often unclear and subject to diverse conditions, limiting planners’ ability to foresee all available resources or use them flexibly.

Disparate budget structures at different levels also contribute to inconsistency and poor coordination across levels. In Burkina Faso, Kenya, Uganda and the Philippines, central governments have shifted towards programme-based budgeting, but these reforms have not spread consistently to subnational governments, which continue to budget using more rigid economic classifications (table 4). The central government in Kenya has attempted to align budget structures across levels by offering guidelines on the programmes to be used by counties, but there remains considerable variation between and across levels in practice.

**Table 4:** Main budget classifications used by central and subnational governments

Country (subnational levels)	Central	Subnational	Health facility
Burkina Faso (commune)	Programme	Economic	Activity based
Indonesia (province and district)	Programme Administrative Economic Functional	Programme Administrative Economic Functional	Programme
Kenya (county)	Programme Economic Some budget lines are cross-cutting (human resources, administrative functions)	Programme Economic	Programme
Mozambique (province and district)	Economic Administrative Functional (programme introduced in 2008 but not yet fully applied)	Economic Administrative Functional (programme introduced in 2008 but not yet fully applied)	Economic Administrative Functional (programme introduced in 2008 but not yet fully applied)
Nigeria (state and LGA)	Economic Administrative	Varies by state Lagos: functional, then organisational, then economic Not consistently aligned with federal budget structure No documentation found for LGAs	(Not clear if local facilities have budgets)
Philippines (LGU)	Programme (Administrative Economic)	Economic with some programmatic elements (varies by LGU)	Programme for Department of Health-owned facilities Economic for local facilities
Uganda (district)	Programme (partial implementation) Economic Administrative Functional	Economic Functional	Economic Activity based

LGA, local government area; LGU, local government unit.

Finally, information system weaknesses limit the effective use of performance data in the budget development process. While lower levels of government are adopting integrated financial management information systems in several study countries, the reforms are nascent. The systems are also not geared towards tracking health spending in a granular way, which hinders their ability to effectively inform future budget allocations.

Policy options to align budget formulation across different levels include enhancing intergovernmental coordination mechanisms and increasing training for subnational government staff to handle the budgeting processes. Furthermore, adopting—and fully implementing—a common programme-based budgeting structure at the subnational level can realign budget priorities across levels while also giving subnational governments more flexibility. The use of financial management information systems in an integrated way across levels can enable greater consistency in budgeting processes.

## Weak subnational expenditure management

Unpredictability and procedural complexity of funding flows to and within subnational governments in the face of limited local revenue contribute to spending volatility, cash flow management problems and low budget execution rates at subnational levels in most study countries, thus also affecting the purchasing function. For example, uncertain and delayed releases from central treasuries have been common in Burkina Faso, Indonesia, Kenya and Uganda, while late or incomplete donor disbursements are an issue in Mozambique and Uganda. In some countries, the way PFM and devolution reforms have been implemented has had the unintended consequence of making spending protocols more complicated and cumbersome in health. Local governments in Nigeria, for instance, can only spend funds after getting approval from states. Likewise, the Ministry of Finance in Uganda imposes strict quarterly expenditure limits that districts cannot exceed.

While local decision-making is theoretically associated with increased transparency and accountability,^8^ this has not been the experience of the study countries. Accounting for the use of funds by subnational governments—both to the central level and to their constituents—remains challenging due to the multiplicity of funding flows, disparate lines of accountability and capacity constraints.^16^ In Uganda, substantial off-budget external funding hinders holistic reporting while in Nigeria’s federal system, there is no central entity that can impose common reporting standards on all levels. Subnational governments do not consistently publish expenditure information in Kenya, Nigeria and the Philippines. Consolidating information about financial and non-financial performance across all levels remains challenging in many of the study countries, limiting accountability for health results.

Reforms to streamline and simplify expenditure management procedures—specifically reducing the number of layers and controllers—can increase the rate of spending and align it with needs. Accelerating digitalisation and improving interoperability of information systems at the central and subnational levels can increase efficiency as well as transparency and accountability between levels and to the public. While these options hold promise for all countries, it seems particularly important for devolved settings where many more actors across different levels of government exercise control over allocation and spending decisions.

## Ineffective subnational purchasing of health services

Subnational governments are purchasers of health services in all study countries in that they allocate funds to frontline facilities in the public sector, like health centres and local hospitals. Purchasing is less explicit in such an arrangement in that subnational governments usually own and manage these same facilities (table 3). In some countries, subnational governments can purchase services from private providers, who represent a large share of service delivery capacity in many settings. Apart from subnational governments, other purchasers such as central health ministries and health insurance agencies also channel funds to the same facilities.

Purchasing is more strategic when decisions over allocations to providers are based on information about population health needs and provider behaviour. In principle, devolution can promote strategic purchasing, as local authorities have more knowledge about local needs. In practice, rigid line-item budget allocations are the dominant mode of payment in all seven countries. Instances of subnational governments using global budgets, capitation or output-based payments for public facilities are rare. Additionally, subnational governments rarely contract private sector providers due to a lack of discretionary funds and capacity constraints. Due to these factors as well as issues of low budget execution and poor expenditure management discussed in the previous section, subnational governments are not spending funds effectively.

Another challenge related to purchasing is lack of autonomy for health facilities; changing how providers are paid only matters if providers can access and use those funds.^3^ Devolving purchasing functions to lower levels of government has not enhanced the financial and managerial autonomy of health facilities in the public sector. In all seven countries, health facilities under the jurisdiction of subnational governments have limited control over financial resources and can rarely participate meaningfully in local planning and budgeting processes. While these facilities generate revenue from national health coverage schemes or user fee reimbursement arrangements, their ability to retain and spend these funds is often limited (table 5). This dilutes the signals that the various purchasers are attempting to send. The problem is most conspicuous in Kenya, Indonesia and the Philippines; payments from national health insurance agencies to many public facilities are diverted to the subnational governments, which continue financing facilities through line-item budgets. Although this problem is not restricted to devolved settings, subnational governments may have a strong interest in limiting facility autonomy to maximise their revenues.^18^

**Table 5:** Flow and use of facility funds

Country	Do public facilities collect user fees? If yes, can they retain and spend them?	If there is national health insurance or other output-based purchasing arrangements, do those reimbursements flow to facilities? Can the facility retain the funds received?	Is revenue generated by health facilities ‘earmarked’ for use in the health sector?
Burkina Faso	Yes, except for family planning and maternal, newborn and child health services covered by Gratuité. These funds are retained and spent by facilities.	A portion of Gratuité payments flows to the health facility bank account to cover operational costs. While hospitals receive money directly in their bank account, payments for primary care facilities are first transferred to districts and then to the facility bank account. Some payments flow to the central medical store for commodities.	Yes. Gratuité reimbursements are also used for health purposes.
Indonesia	Yes for non-JKN members and members who do not use their benefits. Hospitals and some primary care units designated as semiautonomous budgetary units (BLUD) have financial autonomy to retain and spend their revenue. Revenue from non-BLUD primary care units accrues to the local government.	For non-BLUD facilities, JKN payments for public facilities flow to local governments. Public facilities with BLUD status have financial autonomy.	Yes. There are general guidelines for local governments to allocate 10% of their budget to health (but not for salaries). This is not consistently followed. Many local governments use revenue from facilities to meet this threshold.
Kenya	Yes, except health centres and dispensaries. Counties dictate whether hospitals can retain and spend the user fees they collect.	NHIF reimburses facilities under its health insurance programmes as well as other purchasing arrangements (eg, for maternity services). In most counties, hospitals are required to transfer those funds to the county government, while a minority of counties have authorised facilities to retain and spend those funds. Primary care facilities receive less from NHIF but can retain and spend the funds.	It varies across counties, depending on county legislation. Some counties have passed legislation authorising facilities to retain the funds. Others have created a special ‘facility improvement fund’ into which all funds are remitted and then earmarked for use by facilities. In most counties, the funds flow to the county government which can allocate them towards any purpose.
Mozambique	Yes. Facilities remit these funds to the central level, except central and provincial hospitals which can retain 40% of user fees for medicines. Facilities designated as budget units receive their money back. The revenues from health centres and from rural and district hospitals are sent to the district health office.	No national health insurance	Yes, but not necessarily at the level it was generated.
Nigeria	Yes. Whether they can retain and spend these funds varies across states. User fees are not well tracked.	Varies by state	No consistent practice
Philippines	Public hospitals charge fees, while primary care facilities generally do not. DOH-managed hospitals can retain revenue (they are required to use 25% of the funds for upgrading hospital equipment and the remaining for operating expenses and capital outlays). In general, all other LGU-controlled facilities cannot retain their revenue.	Yes, public health facilities can receive and retain PhilHealth reimbursements. But in practice, this varies. In some instances, all funds are remitted to the LGU. On the other hand, some local governments allow their facilities to get their full PhilHealth payments and retain all their income streams.	This varies a lot across LGUs based on whether they allow health facilities to retain any revenue, since all funds remitted to the LGU are treated as general revenue.
Uganda	No, except private wings of hospitals. These funds are remitted to the central level.	Public facilities receive direct grants from the central government for their operating costs. They also receive output-based payments that they can spend following MOH guidance.	Yes, through reprogramming during the annual budget process.

DOH, Department of Health; JKN, Jaminan Kesehatan Nasional; LGU, local government unit; MOH, Ministry of Health; NHIF, National Health Insurance Fund; PhilHealth, Philippine Health Insurance Corporation.

Addressing these challenges calls for a change in the mindset of subnational governments and policy shifts by the central government. Subnational governments must identify as strategic purchasers, which requires greater awareness about this role as well as strengthened capacity in planning, improved allocation of resources to align with population needs and provider performance, and payment methods design. Moreover, central governments can incentivise subnational governments to allocate funds to health providers based on their performance. Finally, changing PFM rules to grant facilities greater autonomy and holding them accountable is important for the success of reforms to make purchasing more strategic,^36 37^ whether the purchaser in question is the local government or a national health coverage scheme (such as health insurance).

## Conclusion

The nature of devolution in a country strongly affects the design, implementation and effects of health financing arrangements and PFM processes in the health sector. The logic of devolution—with more subnational discretion over decision-making and funds—could in principle strengthen the two health financing functions of revenue raising and purchasing. In contrast, shifting the pooling function to lower levels of government leads to fragmentation and is thus less desirable, as health pools should be large and diverse to maximise redistributive capacity. Devolution also leads to the creation of multiple purchasing agencies undermining economies of scale and efficiency objectives. Devolution demands that PFM reforms to streamline health budgeting and expenditure management percolate down to subnational levels of government, which is neither automatic nor easy.

This analysis offers a range of policy options for reconciling decentralisation with health financing and PFM that decision-makers can consider as they design UHC-oriented reforms, though we recognise that their appropriateness and likelihood of success depend on many complex contextual factors. As such, we do not claim generalisability of the findings and policy options across all country settings. Central governments channelling more revenue to subnational governments through earmarked grants is likely the most effective way to increase public financing for health at subnational levels, provided subnational governments cannot offset these increases by reducing their discretionary spending on health. Improving fiscal equalisation through better horizontal revenue sharing arrangements can address regional disparities in spending caused by the fragmented pooling architecture. Here too, earmarked grants for health offer a more direct way to address gaps in per capita health spending compared with block grants. In countries with national health insurance or other health coverage schemes, central government subsidies to cover the poor could redistribute resources from rich to poor regions, although its success depends on several demand-side and supply-side factors.

Strengthening intergovernmental coordination mechanisms and building capacity for budgeting and strategic purchasing can increase spending and improve resource allocation. Moreover, shifting to more flexible budget structures that are aligned across levels can improve budget execution. More spending authority over funds and flexibility in their use can also allow subnational governments to shift to output-oriented payment methods and thus link their allocations to health facilities to population health needs and provider performance. This would help make purchasing more strategic, ultimately improving efficiency, equity in access, financial protection and quality of care.

This exploration of how devolution interacts with health financing and PFM in health points to several topics that warrant further investigation, such as the design of conditional grants to increase local health budgets; strategies for shifting the pooling to higher levels of government to reduce fragmentation; the potential of public subsidies for health coverage schemes to redistribute resources across territories; PFM adjustments to improve subnational governments’ budget execution; the role of digital technologies to enhance visibility over fund flows; strategies to enhance strategic purchasing by subnational governments; and approaches to make pooling and purchasing via national health coverage schemes and subnational governments complementary. Insights about these topics can help countries reconcile the tension between devolution and health financing as well as PFM, in turn accelerating progress towards UHC.

## Data Availability

All data relevant to the study are included in the article.
